# Definition of a Multi-Omics Signature for Esophageal Adenocarcinoma Prognosis Prediction

**DOI:** 10.3390/cancers16152748

**Published:** 2024-08-01

**Authors:** Luca Lambroia, Carola Maria Conca Dioguardi, Simone Puccio, Andrea Pansa, Giorgia Alvisi, Gianluca Basso, Javier Cibella, Federico Simone Colombo, Salvatore Marano, Silvia Basato, Rita Alfieri, Simone Giudici, Carlo Castoro, Clelia Peano

**Affiliations:** 1 Humanitas Research Hospital-IRCCS, 20072 Rozzano, Italy; luca.lambroia@humanitasresearch.it; 2 Human Technopole, 20157 Milan, Italy; carola.conca@fht.org (C.M.C.D.); javier.cibella@fht.org (J.C.); 3 Institute of Genetic and Biomedical Research, National Research Council, UoS of Milan, 20072 Milan, Italy; simone.puccio@humanitasresearch.it; 4 Laboratory of Translational Immunology and Humanitas Flow Cytometry Core, Humanitas Research Hospital, 20072 Milan, Italyfederico.colombo1@policlinico.mi.it (F.S.C.); 5 Upper Gastrointestinal Surgery Unit, IRCCS Humanitas Research Hospital, 20089 Milan, Italy; andrea.pansa@humanitas.it (A.P.);; 6 Genomic Unit, Humanitas Research Hospital, 20072 Milan, Italy; 7 Department of Biomedical Sciences, Humanitas University, 20072 Milan, Italy

**Keywords:** esophageal adenocarcinoma, cancer, immunotherapy, treatment, single-cell RNA, single-cell sequencing, RNA sequencing, transcriptional signature, response to therapy, immune infiltrate

## Abstract

**Simple Summary:**

Esophageal cancer, a highly lethal tumor, contributes to 5% of all cancer deaths, with its primary subtypes being esophageal squamous cell carcinoma (ESCC) and esophageal adenocarcinoma (EAC). While most studies focus on ESCC, this study investigates EAC using single-cell RNA sequencing (scRNA-seq) to analyze CD45^+^ immune cells from tumors and matched non-tumor tissues in therapy-naïve patients. By examining the transcriptional profiles of these immune cells and the entire transcriptome in a cohort of 23 patients, this study identifies distinct transcriptional signatures. These signatures were used to stratify a large cohort of TCGA EAC patients, revealing strong associations with prognosis and clinical outcomes. The findings suggest that these transcriptional profiles can improve prognosis accuracy post-surgery and potentially guide effective therapies, including immunotherapy, for EAC patients.

**Abstract:**

Esophageal cancer is a highly lethal malignancy, representing 5% of all cancer-related deaths. The two main subtypes are esophageal squamous cell carcinoma (ESCC) and esophageal adenocarcinoma (EAC). While most research has focused on ESCC, few studies have analyzed EAC for transcriptional signatures linked to diagnosis or prognosis. In this study, we utilized single-cell RNA sequencing and bulk RNA sequencing to identify specific immune cell types that contribute to anti-tumor responses, as well as differentially expressed genes (DEGs). We have characterized transcriptional signatures, validated against a wide cohort of TCGA patients, that are capable of predicting clinical outcomes and the prognosis of EAC post-surgery with efficacy comparable to the currently accepted prognostic factors. In conclusion, our findings provide insights into the immune landscape and therapeutic targets of EAC, proposing novel immunological biomarkers for predicting prognosis, aiding in patient stratification for post-surgical outcomes, follow-up, and personalized adjuvant therapy decisions.

## 1. Introduction

Esophageal adenocarcinoma (EAC) and esophageal squamous cell carcinoma (ESCC) are among the deadliest cancers in the world, and their incidence is rapidly increasing [1]. In many gastrointestinal cancers, the tumor microenvironment (TME) has been shown to be a prognostic feature and allows the establishment of an “immune core” [2]; however, this approach has not yet been adopted in the management of EAC. While previous studies have shown the important role of tumor-infiltrating lymphocytes (TILs) as a useful predictor for therapeutic response and prognosis in ESCC patients [3], clinicians are still far from effectively predicting the persistence of responses to neoadjuvant co-chemoradiation (CTRT). Improving the prediction of a patient’s response to treatment, tumor progression, and/or recurrence remains a significant challenge. RNA sequencing (RNA-seq) technology has emerged as a powerful tool for the analysis of gene expression in cancer samples, providing a comprehensive view of the transcriptome landscape. By analyzing the RNA expression profiles of cancer samples, RNA-seq is able to reveal novel tissue heterogeneity that can improve patients’ stratification and guide personalized treatment decisions [4]: an example could be the TME and its transcription factors, already associated with tumor development and progression, response to treatment, or antitumor response [5,6,7,8]. Until now, in situ tumor immunology has been acknowledged as highly significant for the prognosis of multiple cancers, even if most of the research in the field of esophageal cancer has focused on ESCC, and marginal attention has been paid to the EAC [8,9]. To bridge this gap, we deeply examined the immune infiltrate of three EAC tumor tissues and their matched non-tumor tissues obtained from three patients who underwent surgery for EAC resection. In parallel, we performed total transcriptome profiling by RNA sequencing on a large cohort of EAC patients to determine the prognosis and other factors related to the clinical course of the disease. Finally, the expression profiles of immune markers and transcriptional signatures identified within our cohort were utilized to stratify a larger cohort of TCGA EAC patients. A strong association with their prognosis was demonstrated, thereby enabling the identification of immunological prognostic biomarkers linked to tumor progression, recurrence, and survival. These findings shed light on the possibility of incorporating immunotherapy strategies, such as immune checkpoint inhibitors and cancer vaccines, into future potential treatment plans for EAC, and emphasize the potential of new cancer treatments to improve patients’ outcomes.

## 2. Materials and Methods

### 2.1. Patients’ Recruitment, Tissue Collection, and Experimental Workflow

After obtaining appropriate consent, a total of 26 patients who underwent surgery for esophageal adenocarcinoma at the Esophagogastric Surgery Unit at IRCCS Istituto Clinico Humanitas from January 2020 to December 2020 were enrolled in this study. For each patient, tumor (T) samples and their matched adjacent tissues (NT) were acquired. Single-cell analyses were performed on patients that did not have any kind of neoadjuvant chemoradiotherapy treatment preceding surgery (untreated patients) to avoid any bias introduced by the therapy; both patients treated with neoadjuvant chemoradiotherapy before surgery (treated patients) and without treatments were chosen for total RNA sequencing analyses. T and NT tissues were processed for single-cell RNA sequencing and total RNA sequencing according to specific protocols. The complete list of patients and their relative clinical data are reported in Supplementary Table S1.

### 2.2. Single-Cell Sequencing: Cells’ Preparation, Library Preparation, and Sequencing

ScRNA-seq of the immune infiltrate in EAC was performed to unravel tissue heterogeneity, enabling a complete definition of all the immune cell subpopulations pervading the tumor site and their gene expression alterations. After surgery, tumor and non-tumor tissues were dissociated to obtain a single-cell suspension with the Tumor Dissociation Kit, human (Miltenyibiotec); cells were then stained with live dead eFluor780 and CD45^+^ antibody and sorted with FacsAria III (BD Biosciences, San Jose, Ca, USA). An average of 7000 cells were loaded into the Chromium controller System (10X Genomics) for gel bead emulsion generation and libraries were prepared using the Single-cell 3’ library preparation approach, according to the manufacturer’s instructions (ChromiumTM Single Cell 3’ Reagent Kits v2-rev C, 10X Genomics, Pleasanton, CA, USA). Libraries were sequenced on the Illumina NextSeq550 platform and an average of 40.000 reads per single cell was obtained.

### 2.3. Analysis of Single-Cell RNA Sequencing Data

The reads obtained from the sequencing of the tumor and non-tumor biopsies were mapped to the reference genome GRCh38 using the CellRanger Software version 3.1.0 (10x Genomics). The raw counts were concatenated and then filtered using the Scater (v1.28.0) [10] and DropletUtils (v1.20.0) [11] packages. We used the emptyDrops function to remove all the droplets with a false discovery rate greater than 0.05, and all cells with a number of UMIs, detected genes or a percentage of mitochondrial and ribosomal genes that were outliers compared to the median absolute deviation. All genes that did not have a minimum of 10 counts in the entire dataset were removed. Cells that were imputed arising from doublets through the doubletCells function were excluded. After the quality filter, cells were analyzed using the Seurat version 4.0.1 package [12,13]. The gene counts of each cell were normalized by dividing them to the library size of their cell; counts were converted in CPM and subsequently log transformed. The cells of the different patients were then further filtered selecting only cells with at least one *PTPRC* gene count and integrated with four patients’ scRNA-seq data from Croft et al. [14] into a single dataset via canonical correlation analysis (CCA) workflow. Subsequent analyses were conducted using only the 2000 most highly variable genes in the dataset. Principal Component Analysis (PCA) was used for dimensionality reduction, selecting the first 15 dimensions for CD45^+^ and the first 40 for CD3^+^ cells, followed by clustering using a graph-based clustering approach [15]; for clustering analysis, the resolution was set at 0.6 for CD45^+^ and 0.7 for CD3^+^ cells. Afterward, Uniform Manifold Approximation and Projection (UMAP) was used for two-dimensional visualization of the resulting clusters. The clusters were annotated by analyzing the expression of a panel of cellular-type marker genes. The T cell sub-population was obtained by selecting the T cell clusters, CD8^+^ and CD4^+^, from the CD45^+^ cell clustering and performing the previous analysis step. Subsequently, the annotated clusters of the T cell subtypes were obtained with the same methodology as those obtained with all the immune infiltrates. The differentially expressed genes among each T cell cluster, extrapolated from tumoral and non-tumoral tissues, were calculated with the normalized count matrix with the MAST algorithm, after the removal of ribosomal and mitochondrial genes. To compare our EAC single-cell RNA data with the publicly available ESCC dataset, we downloaded raw data from GSE145370 [16] and we performed an integrated analysis of the two datasets using the reciprocal-PCA integration workflow of the Seurat Package.

### 2.4. Identification of TF Regulons

Single-cell regulatory network identification was characterized using the Python 3.7.3 package pyscenic [17]. Putative target genes for a supplied list of human transcription factors [18] were identified based on co-expression using the GRNBoost2 algorithm [19]. Co-expression modules were filtered using cis-regulatory motif analysis (RcisTarget) and only modules enriched for putative direct-binding targets of the corresponding transcription factor were retained. Where multiple modules were identified for a TF, these were combined to result in a single regulon per TF. Finally, cells were scored for the activity of each TF regulon using the AUCell algorithm and results were visualized using the clustermap function from the Seaborn Python package.

### 2.5. Polychromatic Flow Cytometry

Frozen samples were thawed in a RPMI-1640 medium supplemented with 10% FBS (Sigma-Aldrich, St. Louis, MO, USA), 1% penicillin-streptomycin and 1% Ultra-glutamine (both from Lonza), and 20 µg/mL DNase I from bovine pancreas (Sigma-Aldrich). After extensive washing with PBS 1× without calcium and magnesium (Sigma-Aldrich), cells were immediately stained with the combination of monoclonal antibodies (mAbs) listed in Supplementary Table S3. Intracellular detection of GZMK, Ki67, CD3, GZMB, and BATF was performed following the fixation of cells with the FoxP3/transcription factor staining buffer set (eBioscience, San Diego, CA, USA) according to the manufacturer’s instructions and by incubating with specific mAbs for 30 min at 4 °C. The flow cytometry procedures for high-dimensional single-cell panel development have been previously described [20]. All data were acquired using the FACS Symphony A5 flow cytometer (BD Biosciences, Franklin Lakes, NJ, USA) equipped with 5 lasers (UV, 350 nm; violet, 405 nm; blue, 488 nm; yellow/green, 561 nm; red, 640 nm; all tuned to 100 mW, except for UV, which was tuned to 60 mW). Flow cytometry data were compensated in FlowJo by using single stained controls (BD Compbeads incubated with fluorescently conjugated antibodies) [21].

### 2.6. Computational Analysis of Flow Cytometry Data

Flow Cytometry Standard (FCS) 3.0 files were imported into FlowJo software (version 9) and analyzed by standard gating to remove aggregates and dead cells and identify CD45^+^ CD3^+^ T cells. A total of 20,000 CD3^+^ T cells per sample were subsequently imported in FlowJo (version 10), biexponentially transformed, and exported for further analysis in Python (version 3.7.3) by a custom-made pipeline of PhenoGraph (v1.5.7) [22] where we modified the Linux-community and the core.py script of PhenoGraph package in order to fix the seed to “123456”). Tumoral and peritumoral samples were labeled with a unique computational barcode for further identification and converted into comma-separated (CSV) files and concatenated in a single matrix by using the merge function of the pandas package. The K value, indicating the number of nearest neighbors identified in the first iteration of the algorithm, was set at 500. The data were then reorganized and saved as new CSV files, one for each cluster, which were further analyzed in FlowJo to determine the frequency of positive cells for each marker and the corresponding median fluorescent intensity (MFI). Subsequent metaclustering of iMFI values was performed using the gplots R package (v3.1.3). UMAP was obtained by UMAP Python package; all scripts mentioned above are available at https://github.com/luglilab/Cytophenograph (accessed on 30 June 2024).

### 2.7. Analysis of Bulk RNA Sequencing Data

RNA was extracted with RNeasy Mini kit (QIAGEN, Hilden, Germany); libraries were prepared with the SMARTer Stranded Total RNA Sample Prep Kit-HI Mammalian (Takara Bio USA, San Jose, CA, USA) and sequenced on the Illumina NextSeq550 platform by generating at least 80 million reads 75 bp paired-end per sample.

The raw reads were mapped against the reference genome GRCh38 with STAR Aligner [23] and the count table was generated using FeatureCounts [24]. Genes with less than 10 raw counts in 1% of the samples or with hypervariable expression were removed before normalization. Normalization was calculated by variance-stabilizing transformation (VST) using the DESeq2 package (v1.40.2) [25]. We excluded all biopsies of tumor tissue that had a Pearson correlation coefficient with their respective tumor biopsy greater than 0.85 from the analysis. Subsequently, surrogate variables that generated non-biological variance among samples were identified. The filtered matrix was used for the differentially expressed gene identification. The surrogate variables identified using the R package “DaMiRseq” [26] were indicated in the design slot of the DESeqDataSet object: in this way, the counts were corrected from the batch effect before the identification of differentially expressed genes. The tumor vs non tumor signature is defined by the differentially expressed genes with a P-adjusted value lower than 0.05. This signature was used for pathway enrichment analysis and to identify potential biomarkers or pharmaceutical targets using the Ingenuity Pathway Analysis (IPA) software (Ingenuity H Systems, www.ingenuity.com). The top 100 upregulated genes outlined by the IPA analysis defined the IPA signature. Finally, to verify whether some of these genes were associated with an early prognosis (progression or relapse of tumor) we repeated the analysis of the RNA-seq data among the tumor biopsies of the patients for whom the early prognosis was known and who had a Pearson coefficient greater than 0.85 with their class, positive or negative early prognosis (Positive or Negative) using only the previously identified upregulated gene counts in tumor biopsies compared to non-tumor biopsies. The resulting differentially expressed genes with a *p*-value lower than 0.05 were selected as early prognosis signatures (EPS).

### 2.8. SODEGIR Analysis

We integrated our total RNA-seq data with CNV data of 87 esophageal adenocarcinoma patients from the TCGA database and PREDA package [27] to verify whether there are genomic regions that are overexpressed or inhibited in our tumoral tissue samples. The matrix of the total RNA-seq counts normalized and corrected with the Damirseq package was used to produce a GE score along chromosomes 1-22 using the statistic option within the PREDA package between the expression values of the T biopsies and the NT biopsies. The average of the CNA values for each gene of the TGCA data was calculated and subsequently the log2 was calculated; finally, these data were used within PREDA to calculate a CN score along the genome. Chromosomal regions showing a GE score above or below a threshold of +/− 0.5 with a q-value < 0.01 and a CN score, according to the GE score, above or below a threshold of +/− 0.1 with a q-value < 0.01 were classified, respectively, as GAIN or LOSS.

### 2.9. Survival Analysis

The IPA signature, the EPS, and the 37 genes of the DEGs that fall into GAIN regions were used to verify the existence of an association between these and the prognosis of 78 patients with esophageal adenocarcinoma. The ESCA court of TCGA, using RNA-seq data and the present clinical information, were used to construct Kaplan-Meier curves through the survival package (v3.5-7) [28] and survminer R package (v0.4.9).

For each signature, a score was obtained for each of the 78 patients who were divided into two groups based on a threshold. For bulk RNA-seq, the threshold to separate the patients in two cohorts was chosen between the first (25% of the patients) and the third quartile (75%). For scRNA-seq signatures, the patients were splitted by median of the score signatures. The signature scores were calculated as the average of the logTPMs of the signature gene counts.

Furthermore, the association between the survival of the patients and their content of cell types, that we found in our single-cell analysis, was analyzed. The bulk RNA-seq data from the 78 TCGA patients were normalized for the T cell content to evaluate the effective impact of our scRNA-seq signature. Then, the top 50 genes differentially expressed by each cell type were used to estimate the cell type content in the bulk RNA-seq data of each sample (Supplementary Table S2). 

The *p*-values for all curves were calculated with the log-rank test. The EPS and single-cell signatures were tested as overall survival across 30 months, the top 100 IPA biomarker DEGsS and DEGsS in GAIN regions were tested across 60 months. For the correlation between the survival and clinical parameters of patients with signatures, the TNM parameters, when available for the patient, were reorganized as follows: staging N and M other than N0 and M0 were all merged into N1 and M1; for the T staging, the pairs T1 and T2 and T3 and T4 were merged with each other; and the age of the patients was divided into two categories based on whether the patients were over or under 65. The R survminer package was used for the cox regressions and for the chi-square tests and for the odd ratios, the function oddratio.fisher from the R epitools package was used. The association between the age of the patients and the signatures was tested with the wilcox.test by the compare_means function of the ggpubr package.

## 3. Results

### 3.1. Single-Cell Level Analysis of Esophageal Adenocarcinoma Immune Infiltrate

**Figure 1:**
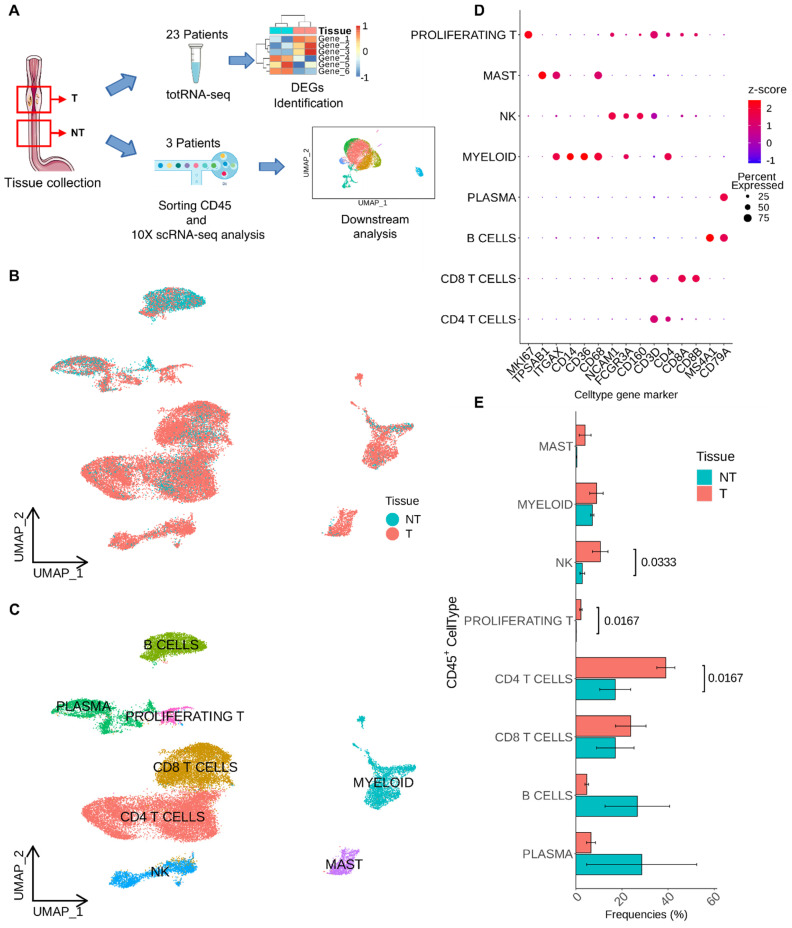
CD45^+^ cells annotation. (**A**) Schematic representation of the experimental workflow. (**B**) UMAP visualization of CD45^+^ clusters according to their tissue of provenance. (**C**) UMAP visualization of annotated CD45^+^ cell clusters. Annotations were made considering the differential expression of the main cell type gene markers. (**D**) DotPlot of the expression level of gene markers specific for each cell type. (**E**) Barplots of the relative abundance of cell clusters according to their tissue of provenance; the bars represent the mean of the frequencies while the error bar represents the standard deviation; *p*-values were computed by Mann-Whitney U test.

For scRNA-seq experiments, the entire workflow of our study is shown in Figure 1A, tumor (T) and matched non-tumor (NT) biopsies from three patients who did not receive any pharmacological treatment were collected and analyzed. We then integrated our data with scRNA-seq data from Croft et al. [14], selecting only CD45^+^ cells, including four treatment-naïve patients for a total of seven patients analyzed to obtain a larger cohort of EAC. Uniform Manifold Approximation and Projection (UMAP) of T and NT EAC immune cells outlined a differential enrichment of those cells according to the tissue of origin (Figure 1B) and bioinformatic analysis was able to define eight clusters of CD45^+^ cells. In detail, EAC immune infiltrate was composed of myeloid, mast, plasma, NK, B, CD8^+^ T, and CD4^+^ T cells (Figure 1C). The dot plot in Figure 1D shows the expression of marker genes used for the cell type annotation.

When comparing the percentage frequencies of CD45^+^ cells across EAC samples based on their tissue of origin, we observed that tumor samples generally exhibited an enrichment of T-infiltrating lymphocytes (TILs), specifically CD4^+^ T cells and NK cells, compared to non-tumoral samples (Figure 1E). Furthermore, NT tissues showed a higher presence of B cells and CD8^+^ T cells, whereas T tissue resulted in enrichment in the other identified CD45^+^ cell types. To assess whether the different subsets of T cells could be differentiated according to a specific transcriptional program, we employed python single-cell regulatory network inference and clustering (pySCENIC) analysis on our single-cell dataset for CD8^+^ and CD4^+^ cells infiltrating tumor tissue. This analysis revealed several active regulons that confirm the differentiation and activation of these cell types (Supplementary Figure S1). Additionally, we integrated the dataset of the seven EAC patients with the scRNA-seq data of ESCC from Zheng et al. [16]. We identified the same cell types reported in Supplementary Figure S2A, albeit with differences in the relative abundances between the two types of esophageal cancer (Supplementary Figure S2B). In particular, EAC seems to be characterized by a greater number of tumor-infiltrating CD4^+^ T cells, while ESCC shows a marked enrichment of myeloid cells.

### 3.2. Dissection of T Cells’ Heterogeneity in Esophageal Adenocarcinoma

**Figure 2:**
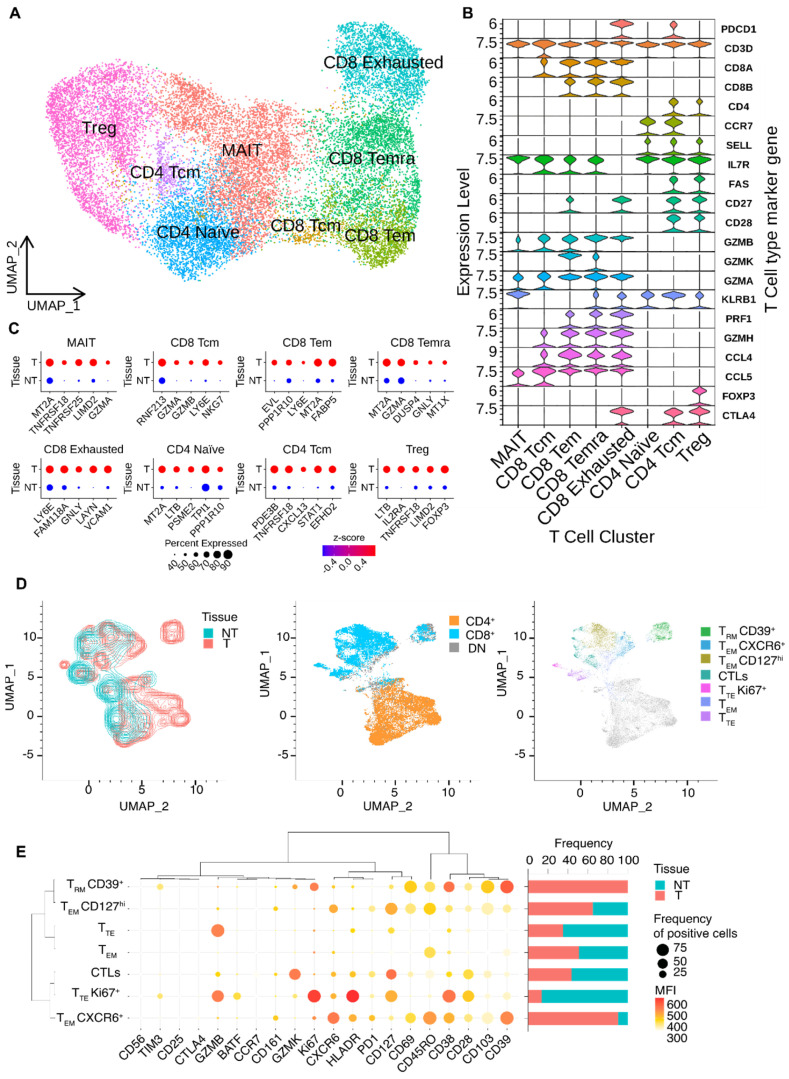
T cell annotation and differential expression analyses. (**A**) UMAP visualization of annotated T cell clusters. (**B**) Violin plot with the average expression of cell type marker genes used for annotation. (**C**) Differential gene expression in each T cell subcluster comparing tumor and non-tumor samples. (**D**) UMAP analyses of the separation of the cells according to the tissue type (**left panel**), the T cell type (**central panel**), and the annotation of each subcluster (**right panel**). (**E**) Dot plot showing the cluster identification according to the MFI of the antibody, the frequency of positive cells (**left panel**), and the frequency of each cell population according to the tissue of origin (tumor or non-tumor tissue, **right panel**). Each cluster was identified considering the mean fluorescence intensity (MFI) of the antibody and its frequency in each tissue type.

The heterogeneity of the T cells’ cluster was explored by reanalyzing the subset of CD45^+^ CD3^+^ cells. As depicted in Figure 2A, the sub clustering of only T cells revealed eight distinct clusters, which were manually annotated based on their gene marker expression (Figure 2B). The markers’ genes for each cell type were chosen among the differentially expressed genes (DEGs) identified through researching the literature. The DEGs among CD45^+^ cells originating from tumor tissue are reported in Supplementary Table S2. We outlined three clusters with higher frequency in tumor tissues (Supplementary Figure S3): T regulatory (Treg) cells, Mucosal Associated Invariant T (MAIT) and exhausted CD8^+^ cells. In contrast, Temra CD8^+^ were more abundant in NT tissues. CD4^+^ naïve, CD4^+^ Tcm, CD8^+^ Tcm and CD8^+^ Tem cells showed no differences in abundance between tumor and non-tumor tissues. With reference to annotation, the CD8^+^ Tem cluster showed high expression of cytotoxic markers such as granzyme K (*GZMK*), granzyme A (*GZMA*), granzyme B (*GZMB*), granzyme H (*GZMH*), and perforin 1 (*PRF1*), but low expression level of *CCR7*. Naïve CD4^+^ T cells were characterized by the expression of *IL7R* and the T cell differentiation markers *SELL* and *CCR7*. The subset of cells expressing *CCR7*, *SELL*, and FAS was defined as CD4^+^ Tcm. Treg cluster was characterized by the expression of CD4, CD25 (*IL2RA*), *BATF*, and *FOXP3*. Cells within the CD8^+^ Temra cluster were also found to be widely distributed in NT tissue; they exhibited enrichment in cytotoxic markers including *PRF1*, *GZMA*, *GZMB*, *GZMH*, although with low expression of the CD8^+^ Tem gene marker *GZMK*. Additionally, these cells also expressed inflammatory markers like *CCL5* and *CCL4*. CD8^+^ cells with lower expression of cytotoxic markers but higher levels of *IL7R* were annotated as CD8^+^ Tcm. The MAIT cluster was defined by the expression of the cytotoxicity markers CD61 (*KLRB1*), *IL7R*, and *CCL5*. We then analyzed the highly differentially expressed transcripts between tumor and non-tumor tissue in each CD8^+^ T cell subpopulation (Supplementary Table S2). A strong fold change in the average expression level of the main differentially expressed gene (DEG) markers of each CD3^+^ cell cluster was observed when comparing tumor and non-tumor tissues (Figure 2C). As expected, CD8^+^ T cell-infiltrating tumor tissues showed high levels of cytotoxic markers and metallothionein, which are involved in maintaining homeostasis and regulating apoptotic and autophagy pathways. Interestingly, at the gene expression level, TILs from tumor and non-tumor tissues displayed distinct profiles. This allowed us to define a differential transcriptome profile signature for each CD8^+^ subcluster in tumor and non-tumor tissue. We then focused our analysis on TILs within the tumor tissues. After defining their specific transcriptional signature, we used it for subsequent analyses. To validate our findings with a protein-based approach, we designed a 22-parameter polychromatic flow cytometry panel using the signature markers previously identified from our single-cell analysis. This panel was equipped to detect markers of activation (CD38, CD45RO, CD127, HLA-DR), exhaustion (CD39), proliferation (KI67), and metabolic activity (GZMB, GZMK), which were identified at the transcriptomic level by the single-cell sequencing analysis in both tumoral and non-tumoral tissues. This flow cytometry panel was specifically designed to be representative of the cell clusters we previously described and outlined in Figure 2A. Figure 2E shows the differential expression of the markers used to identify these clusters. The UMAP shows the dimensional reduction in the cells according to the tissue type (Figure 2D, left panel) and the CD8^+^ or CD4^+^ T cell phenotype (Figure 2E, central panel). We then focused our attention on CD8^+^ T cells: using PhenoGraph, we identified seven different CD8^+^ clusters (Figure 2D, right panel).

Among the memory T cell subsets, we distinguished between resident and effector cells. TRM cells showed high expression of residency markers such as CD39^+^ and CD103^+^, and variable levels of the proliferation marker KI67^+^. Interestingly, this subset appeared to be exclusive to tumor tissue. The remaining effector cells were characterized by the memory marker CD45RO. Notably, two subclusters expressed the tissue residency marker CD69, and were labeled as CD127hi and CXCR6^+^. The CD127hi was characterized by high expression of the differentiation marker CD127, encoded by the Interleukin 7 receptor (IL7Ra). The CXCR6^+^ subset exhibited high levels of the exhaustion marker CD39 along with variable levels of the activation marker CD38, being more prevalent in tumor cells. The effector subset consisted of two clusters: TTE and TTEKI67^+^. Both expressed high levels of the effector molecule GZMB, but TTEKI67^+^ also displayed high expression of KI67 and the activation markers HLA-DR and CD38. At last, we identified CTLs cells with a cytotoxic phenotype characterized by GZMK expression and the effector differentiation marker CD127. Detailed information about the antibodies used for the panel can be found in Supplementary Table S3.

We then integrated our EAC dataset with a public ESCC dataset to investigate the potential similarities among the TILs of the two types of esophageal cancer. The distribution of EAC CD45^+^ cells mostly overlapped with the one of ESCC. As expected, the abundance of CD45^+^ cells differed due to the diverse sample sizes and cancer types (Supplementary Figure S2A). Additionally, UMAP identified 12 clusters using a panel of markers selected from the highly differentially expressed genes, highlighting the similarity of CD45^+^ cells between EAC and ESCC (Supplementary Figure S2B). The complete list of genes used for the annotation is provided in Supplementary Figure S3. Bar plots showing the differential composition of EAC and ESCC tissues are presented in Supplementary Figure S2C.

### 3.3. Whole-Transcriptome Profiling of Esophageal Adenocarcinoma Tissues for the Identification of a Prognostic Signature 

**Figure 3:**
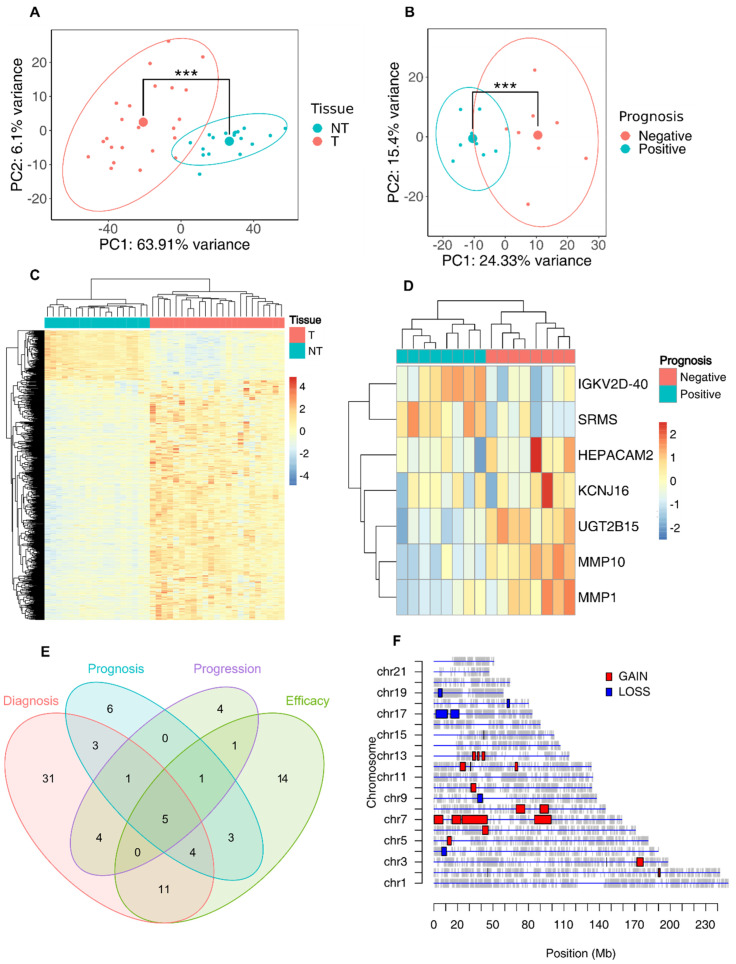
Total RNA expression analysis of tumor and non-tumor esophageal tissues. (**A**) PCA of bulk RNA-seq samples visualized according to the tissue of origin (*** *p*-value < 0.001). (**B**) Heatmap of differentially expressed genes from total RNA sequencing data comparing tumor and non-tumor samples (*** *p*-value < 0.001). (**C**) PCA of bulk RNA-seq samples visualized according to the early prognosis. Patients with a bad prognosis could have had either progression or relapse of the tumor. (**D**) Heatmap of differentially expressed genes according to early prognosis data. (**E**) IPA analysis of the differentially expressed genes in tumor samples after bulk analysis showing the annotated biomarkers among the top 100 upregulated DEGs. (**F**) Plot showing GAINs and LOSSes in genomic regions obtained by SODEGIR analysis of total RNA-seq data integrated with CNV data from TGCA database.

Total RNA sequencing was performed on a wider cohort of patients compared to the one used for single-cell sequencing. For this task, we also included patients who had undergone neoadjuvant therapies before surgery. In total, RNA from 55 tissue samples was extracted and subsequently sequenced. Principal Components Analysis (PCA) confirmed the segregation of the samples according to the tissue of origin: PC1 separated tumor and non-tumor tissues, explaining the 63.91% of the variance. The distance measurement of the centroids yielded a statistically significant *p*-value (*p*-value < 0.001) (Figure 3A). In Figure 3B, a hierarchically clustered heatmap shows the topmost significant DEGs between tumor and non-tumor samples, indicating a clear separation in the expression profiles of the two tissue types. We then investigated whether a specific transcriptional profile could be associated with an early prognosis of EAC. Figure 3C shows the PCA plot with a significant separation of the samples according to the postoperative course (*p*-value < 0.001).

In Figure 3D, the top differentially expressed genes between positive and negative postoperative courses are shown in a hierarchically clustered heatmap, revealing a clear separation between the two groups and identifying a specific transcriptional profile associated with relapse/progression. Ingenuity Pathway Analysis (IPA) of upregulated genes in EAC tumor samples was then performed to evaluate the enrichment of markers involved in tumor onset and/or progression or known drug targets. As shown in Figure 3E, a wide panel of gene markers associated with diagnosis, disease progression, low drug efficacy, poor prognosis, or low response to therapies was outlined. As expected, pathway enrichment analysis of genes upregulated in tumor samples revealed enrichment in the pathways involved in tumorigenesis (Supplementary Figure S4). Finally, we integrated our total RNA sequencing data with Copy Number Variation (CNV) data from the TCGA database using SODEGIR analysis, highlighting regions of chromosomal instability previously associated with esophageal cancer [29,30,31] (Figure 3F). Then, we merged data from SODEGIR and IPA analyses, and identified seven genes (*TREM1*, *PGC*, *AGR2*, *AGR3*, *SFRP4*, *INHBA* and *COL4A1*) that were already recognized as IPA biomarkers (Supplementary Table S4). The upregulated DEGs, which were located inside the GAIN genomic regions, were used to construct a signature used for subsequent analyses.

### 3.4. Association between the Prognostic Signatures and Patients’ Survival

**Figure 4:**
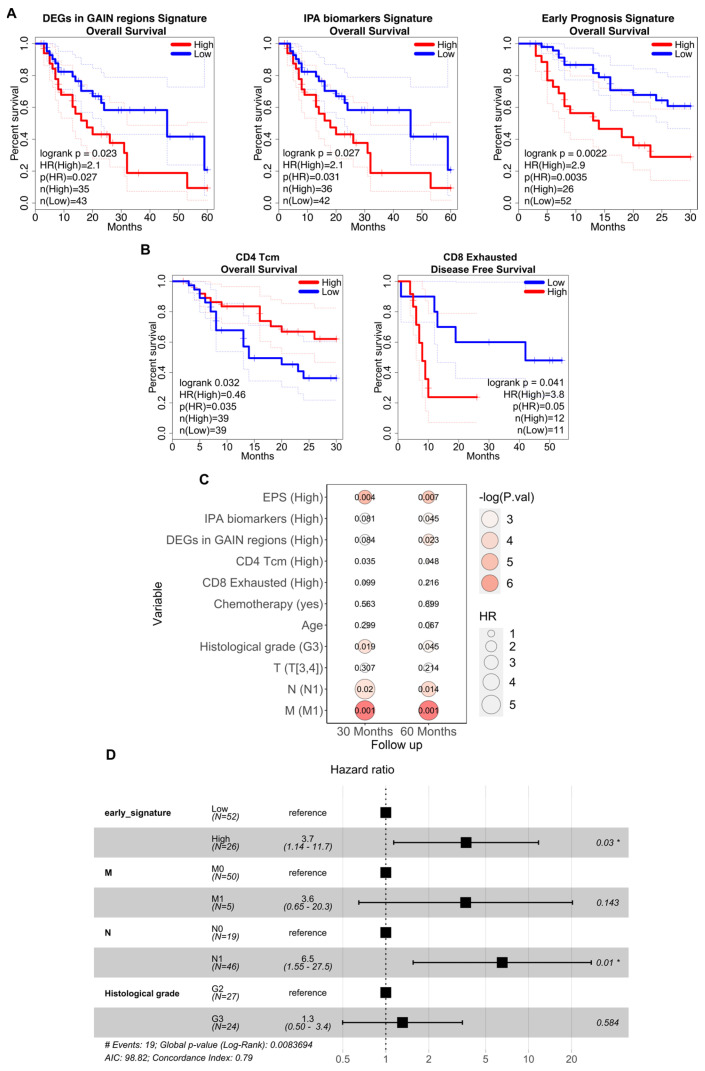
Survival Kaplan-Meier curves of TCGA EAC patients. (**A**) The first two plots show the Kaplan-Meier curves with overall survival of TGCA EAC patients at 60 months separated according to their values of expression of the PREDA signature score, to the values of the DEGs signature reported from IPA as the biomarkers’ signature score. The last plot shows overall survival of TGCA EAC patients at 30 months separated according to their values of expression of the EPS score. (**B**) Kaplan-Meier curves with the overall survival of TGCA EAC patients at 30 months separated according to their values of expression of the CD4 Tcm cells signature (on the left) and the disease-free survival ones of our cohort of total RNA patients separated according to the CD8 exhausted expression score. *p*-values were calculated using the log-rank test. (**C**) Univariate Cox proportional hazards regression between the signatures, the main available clinical parameters used for the diagnosis of EAC, and the OS of TCGA patients at 30 or 60 months of follow-up; the size of the dots reflects the hazard ratio, the color represents the -log(*p*-value). (**D**) Multivariate Cox regression analysis of early prognosis signature with M, N and histological grade parameters (* *p*-value < 0.05).

We used the transcriptional signatures derived from our data analysis to predict survival in a larger and different cohort of EAC patients. Total RNA-seq data from tumoral biopsies of 78 EAC patients from the TCGA database were obtained, and survival prediction was performed. We tested whether the differential signatures could separate TCGA patients into two groups: one with low expression and one with high expression of the signatures. We then assessed if the expression levels of the signatures were associated with different prognoses. We specifically examined the association of DEGs in GAIN genomic regions, DEGs related to the IPA signature, and those specific to the early prognosis signature (EPS), with the overall patient survival (Figure 4A). In all three analyses, the separation of patients according to their prognosis was statistically significant (*p*-values = 0.023, 0.031, 0.002, respectively). Patients with higher overall survival probability (up to 60 months post-surgery) and, thus, a good prognosis, exhibited low expression levels of both DEGs in GAIN genomic regions and IPA biomarkers. Conversely, patients showing high expression levels of these differential signatures had a lower overall survival probability, indicating a poor prognosis. Similarly, high expression of EPS was negatively associated with patient prognosis within the first 30 months post-surgery. Next, we examined survival curves using the signatures of the T cell subtypes identified in our scRNA-seq analysis, focusing only on the T cells infiltrating tumor tissues (Figure 4B). Patients with high expression of the CD4^+^ Tcm cluster signatures had a good prognosis, showing a high overall survival probability in the first 30 months post-surgery. Additionally, exhausted CD8^+^ T cells were able to stratify patients in our cohort (analyzed with total RNA-seq) for disease-free survival (DFS) data, suggesting a potential link to treatment success in preventing relapse. In particular, high expression of these cells seemed to be associated with a poor response to treatments.

Finally, we investigated whether the transcriptomic signatures derived from this study could predict patient prognosis alongside established clinical parameters in EAC diagnosis, such as the TNM staging system and the histological grade of the tumor. Initially, univariate Cox regression estimated the hazard ratios (HR) for each parameter independently, revealing correlations and a substantial increase in HR with survival at 30 and 60 months only for TNM N and M factors, and histological grade (Figure 4C). Subsequently, these factors alongside each expression signature (excluding TNM M to ensure sufficient event numbers across cohorts over time) were included in a multivariate Cox regression analysis. Among the signatures, only EPS maintained an association with a poorer prognosis after adjusting for the other factors (Figure 4D). To understand the type of correlation with clinical parameters, odds ratios and chi-square tests were performed, demonstrating independence from clinical parameters through the distinct associations of the signatures (Supplementary Figure S5). Additionally, the Wilcoxon test showed no age-related differences between low and high signature cohorts.

## 4. Discussion

In this study, we conducted single-cell sequencing and total RNA sequencing analyses on tumor and matched non-tumor tissues from patients with esophageal adenocarcinoma (EAC) to characterize immune cell subpopulations and identify gene markers associated with patients’ clinical outcomes. CD45^+^ cells infiltrating tumor tissues were analyzed at the single-cell level, resulting in the identification of eight distinct subpopulations. Further analysis of T cells revealed eight subclusters with differential gene expression between tumor and non-tumor tissues.

Bulk total RNA-seq analysis across a larger cohort of patients clearly distinguished between tumor and non-tumor tissues. Transcriptome analysis focused on tumor samples enabled stratification of patients based on early prognosis, outlining a panel of DEGs linked to clinical outcomes. Notably, *UGT2B15*, previously associated with pathogenesis and prognosis of gastric cancer [32,33] and *HEPACAM2*, upregulated in patients with poor prognosis and linked to metastasis in various types of cancer, were highlighted. MMPs, including *MMP-1* and *MMP-10*, known for their roles in esophageal tumorigenesis, were also found to be upregulated in tumor samples [34,35]. Conversely, we observed downregulation of *IGKV2D-40*, part of an immune-related gene panel for colorectal cancer prognosis.

We performed gene set enrichment and SODEGIR analyses to identify enrichment in *TREM1*, *PGC*, *INHBA*, and AGR, all of which are involved in cancer-related pathways and associated with patient prognosis in esophageal carcinomas and premalignant Barrett’s epithelium [36].

Analysis of EAC patients from the TCGA dataset showed that high expression of genes located within genomic GAIN regions, as outlined by SODEGIR analysis, correlated with poor prognosis (Figure 4A). This is consistent with previous studies linking genomic alterations in EAC to tumor malignancy.

Through gene set enrichment analysis, we identified potential prognostic biomarkers in EAC, suggesting their utility in predicting patient outcomes. To further validate our findings, we tested this signature on the TCGA dataset: patients with low expression of the identified DEGs had a better prognosis within the first 30 months of follow-up. Additionally, we observed that elevated expression levels of the CD4^+^ Tcm cluster signature and reduced expression of the exhausted CD8^+^ cluster signature reflected differences in the tumor immune infiltrate composition and were associated with positive treatment outcomes.

We speculate that these immune cell types contribute to the anti-tumor responses, and the identified differential expression signatures could be used to develop a cytofluorimetric panel for early detection and the prediction of tumors which are likely to respond favorably. Overall, our study delineates immune cell subpopulations pervading at the EAC tumor site and their gene expression profiles, providing insights into the EAC immune landscape and potential therapeutic targets. Furthermore, these results propose the potential role of novel immunological biomarkers for predicting EAC prognosis, aiding in the stratification of the patients for post-surgical outcomes and follow-up or guiding the design of personalized follow-up programs and decisions regarding adjuvant therapies.

## 5. Conclusions

In conclusion, this study provides a detailed characterization of immune cell subpopulations and their gene expression profiles within esophageal adenocarcinoma (EAC) tissues. The identification of specific transcriptional signatures and differentially expressed genes linked to clinical outcomes underscores their potential as prognostic biomarkers. These findings enhance our understanding of the EAC immune landscape and highlight novel therapeutic targets. The results also propose new immunological biomarkers that can predict patient prognosis, assist in post-surgical stratification, and inform personalized follow-up and adjuvant therapy decisions.

## Data Availability

The raw data of scRNA-seq and total RNA sequencing analysis performed in this study are available at https://zenodo.org/record/7898240#.ZFSZbs5ByN4 (accessed on 30 June 2024) and on Gene Expression Omnibus repository, BioProject ID: PRJNA1141373.

## Supplementary Materials

The following supporting information can be downloaded at https://www.mdpi.com/article/10.3390/cancers16152748/s1, Figure S1: Distinct transcriptional regulation circuits for TILs in EAC T cells. Figure S2: scRNA integration with ESCC. Figure S3: CD3^+^ Cells subcluster proportion. Figure S3: CD3^+^ Cells subcluster proportion. Figure S5: Correlation study between transcriptional signatures and clinical parameters of TCGA patients. Table S1: Patient information, origin, morphology, grading, treatments and follow up information of EAC patients evaluated in the current study. Table S2: Differentially expressed genes specific in tumor tissue among the 14 clusters identified by scRNA-seq. Table S3: List of all antibodies for flow cytometry used in this study. Table S4: List of all the genes found to have gain or loss in CNV with SODEGIR analysis.
