# Supplementary material for: Definition of a Multi-Omics Signature for Esophageal Adenocarcinoma Prognosis Prediction
Source: Cancers (Basel). 2024 Aug 1;16(15):2748. doi: 10.3390/cancers16152748 (PMC11311406; doi:10.3390/cancers16152748)
Supplement: Supplementary file 1 [file cancers-16-02748-s001.zip › cancers-3107018-supplementary.pdf]

Supplementary material: "High-dimensional Single-Cell Immunomonitoring for Esophageal Adenocarcinoma prevention and treatment"

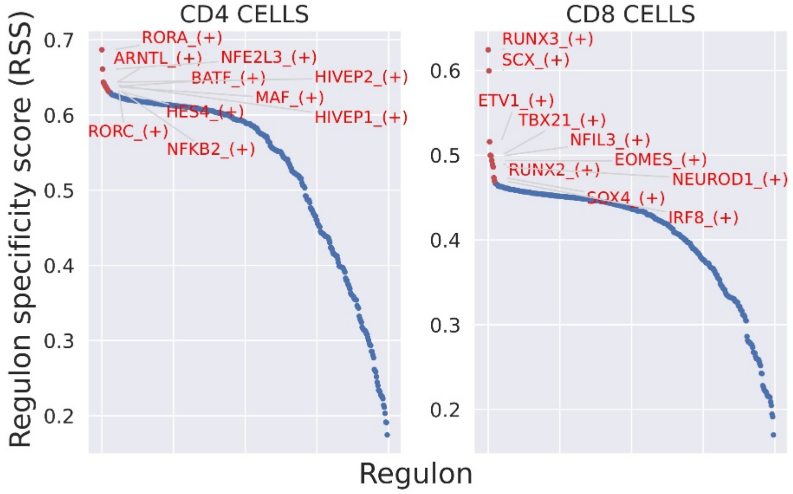

Supplementary Figure S1. Distinct transcriptional regulation circuits for TILs in EAC T cells.
Dot plot showing the enrichment analysis of transcription factor binding motif of cluster obtained from scRNA-seq data. In the Y axis is shown the Regulon Specificity Score (RSS), the red dots indicate the top 10 most enriched regulome.

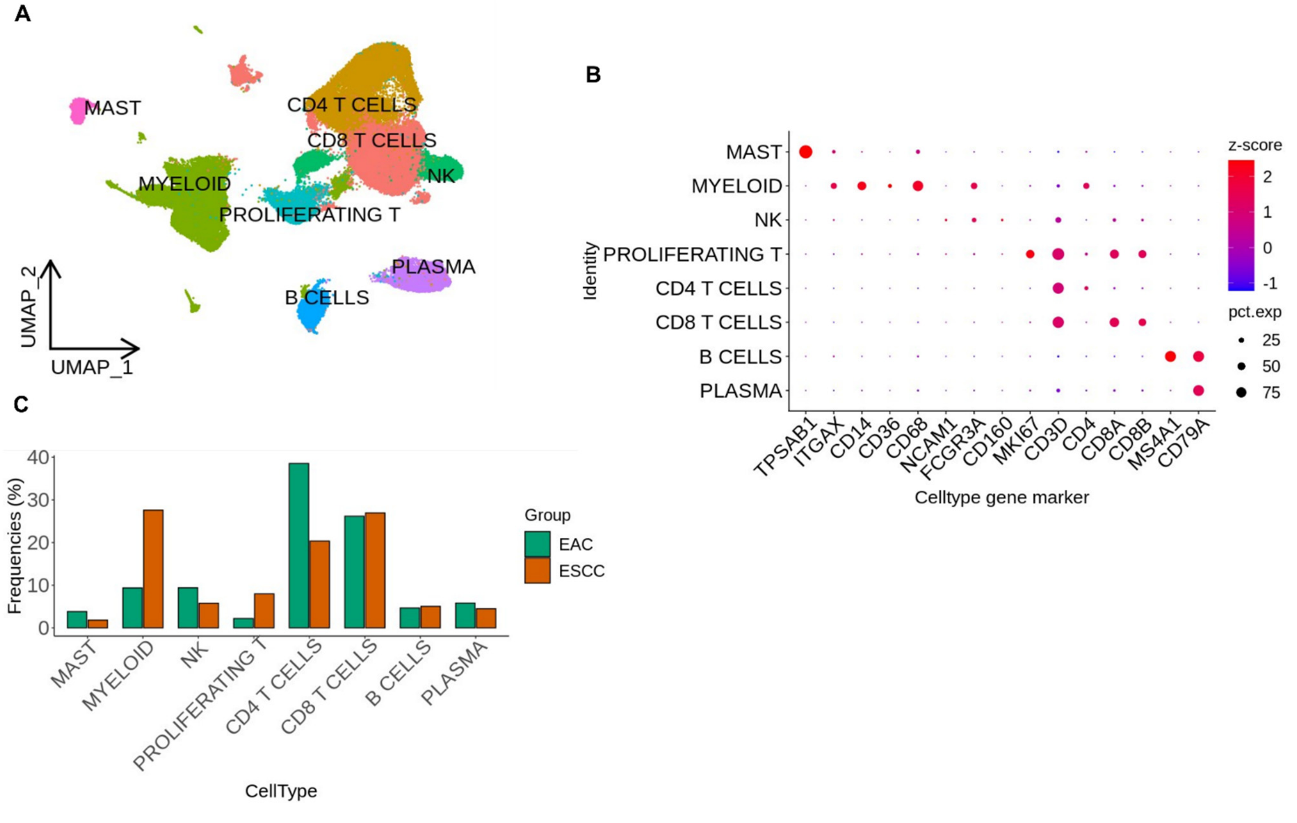

Supplementary Figure S2. sc-RNA integration with ESCC.
A) UMAP plot labeled by cluster annotation of integrated scRNA dataset of EAC and ESCC data. B) expression of cell type marker genes used for cluster annotation of the integrated dataset. C) Frequency barplots showing the relative composition of clusters according to tissue of provenance in ESCC and EAC.

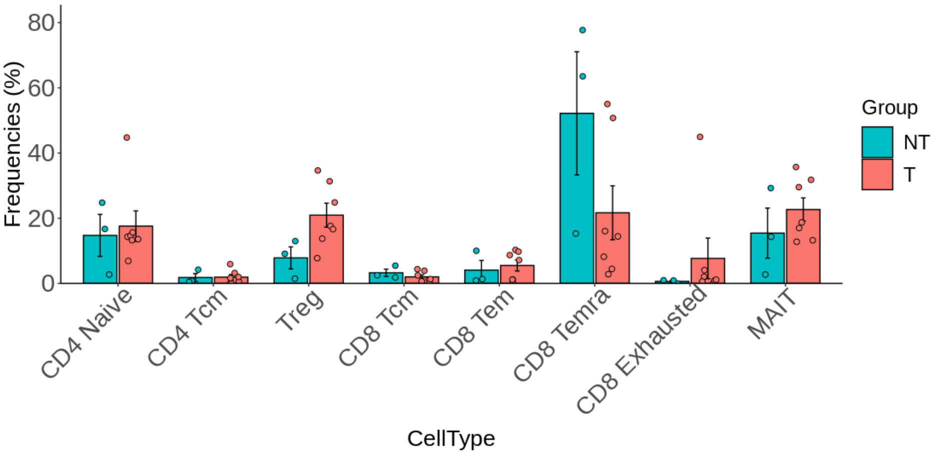

Supplementary Figure S3. ***CD3*****⁺ Cells subcluster proportion.**

Barplots showing the relative composition of *CD3*⁺ Cells subtype in Tumoral (T) and Non Tumoral (NT) areas; each point is the percentage of subtype in a sample, the bar represents the mean of the frequencies while the errorbar the standard deviation.

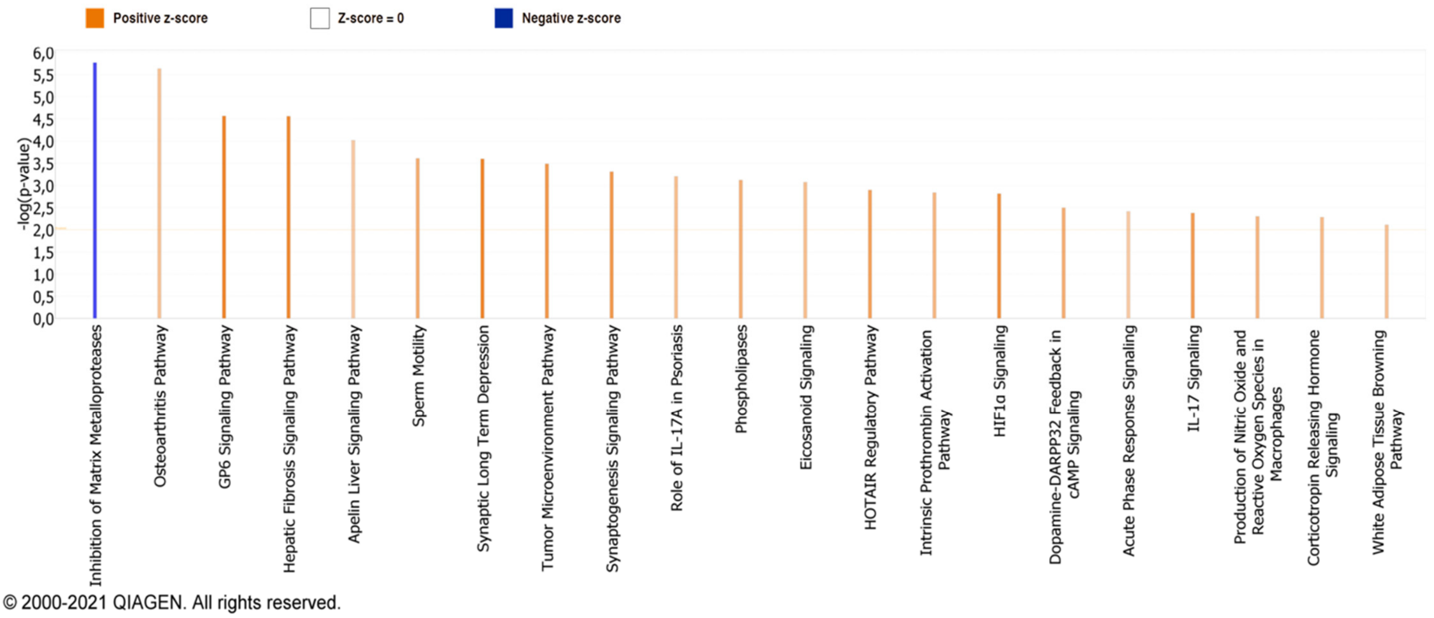

Supplementary Figure S4. Pathway analysis T vs NT: pathway enrichment made by Ingenuity Pathway Analysis (IPA) software with the differential expressed gene of totalRNA bulk analysis. In the figure are shown the pathways with a p-value<0.01 that IPA found enriched in tumor samples. Colors represent the Z-score: blue = negative value of Z-score; red = positive value of Z-score.

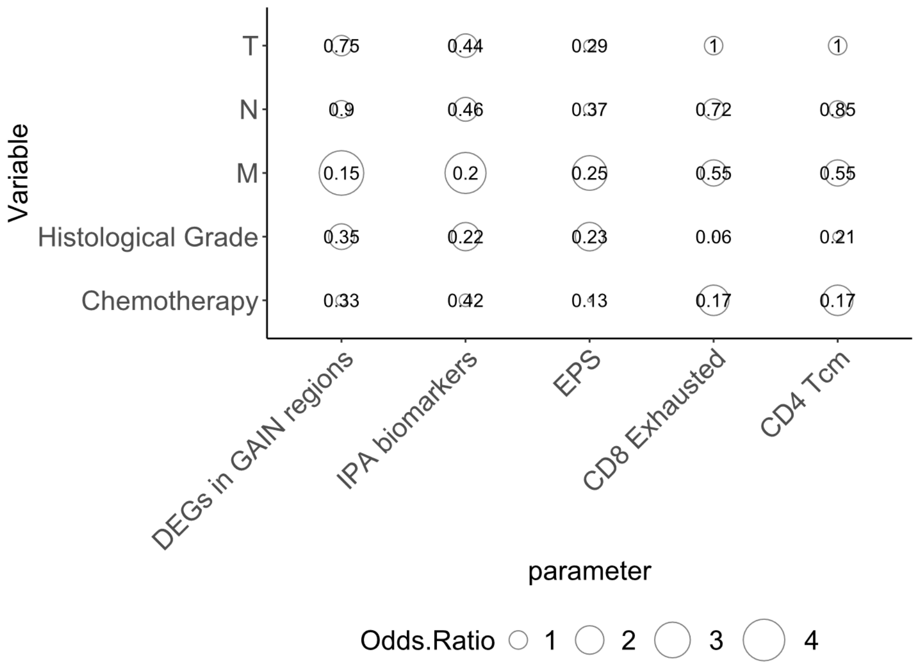

Supplementary Figure S5. Correlation study between transcriptional signatures and clinical parameters of TCGA patients: dotplot with p-values obtained performing Pearson chi-square test between signatures and clinical parameters; the size of the dots reflects the respective value of the odd ratio.

Supplementary Table S1. Patient information, origin, morphology, grading, treatments and follow up information of EAC patients evaluated in the current study. Y=YES, N=NO. HP: presence or absence of Helicobacter Pylory infection; Response: Index of tumoral regression (Becker); PPI: Use of proton pump inhibitors; Postoperative events: relapse (R) or Progression (P); The grade is n/a due to the effect of the therapy.

Patient ID	Gender	EAC type	Metaplasia	Neoadj therapy	response (Becker)	Barrett	HP	PPI	grade	Lymph. inv	TNM	follow up (months)	Post operative events
Patient _01	Male	cardias	Y	Y	2	N	Y	Y	G2	1	ypT3N0 R0	10	R
Patient _02	Male	cardias	N	Y	3	N	N	Y	G3	1	ypT3N1 R0	7	P
Patient _03	Male	Distal	N	Y	3	N	N	N	G2	0	ypT2N0 R0	10	0
Patient _04	Male	distal	Y	Y	3	Y	N	Y	G2	0	ypT2N0 R0	13	0
Patient _05	Male	distal	Y	Y	2	N	N	Y	G3	1	ypT3N3 R0	12	R
Patient _06	Female	distal	N	Y	2	N	N	N	G3	1	ypT3N3 R0	9	R
Patient _07	Male	distal	N	Y	1b	N	N	N	n/a	1	ypT3N1 R0	0	-
Patient _08	Female	cardias	N	Y	3	N	N	Y	G2	1	ypT3N3 R0	13	P
Patient _09	Female	cardias	Y	N	-	N	N	Y	G2	1	pT3N3a R0	5	P
Patient _10	Female	distal	N	N	-	N	N	N	G2	1	pT3N0 R0	1	-
Patient _11	Male	cardias	Y	Y	3	N	N	Y	n/a	1	ypT3N1 R0	19	P
Patient _12	Male	cardias	N	Y	3	N	N	N	G3	1	ypT3N0 R0	54	0
Patient _13	Male	distal	N	Y	2	N	N	Y	n/a	1	ypT3N3 R0	42	P
Patient _14	Male	distal	N	Y	3	N	N	Y	G3	1	ypT3N1 R0	4	P
Patient _15	Female	distal	N	Y	4	N	N	Y	G3	1	ypT3N0 R0	5	0

Patient _16	Male	distal	N	Y	2	N	N	N	G3	1	ypT3N2 R0	6	P
Patient _17	Male	distal	Y	N	-	Y	N	Y	G2	0	T1aN0 R0	10	0
Patient _18	Female	distal	Y	Y	1a	N	N	Y	G2	0	ypT0N0 R0	45	0
Patient _19	Male	cardias	N	Y	2	N	N	Y	G3	0	ypT2N1 R0	26	0
Patient _20	Male	cardias	N	N	-	N	N	Y	G2	0	pT2N0 R0	5	0
Patient _21	Male	distal	N	N	-	N	N	Y	G3	1	pT3N1 R0	8	R
Patient _22	Male	cardias	N	Y	1b	N	N	N	G3	0	ypT1bN0 R0	30	0
Patient _23	Male	distal	N	Y	3	N	N	N	G2	1	ypT3N0 R0	51	0
Patient _24	Male	distal	N	Y	2	N	N	Y	G3	1	ypT3N3 R0	1	P
Patient _25	Male	cardias	N	N	-	N	N	Y	G2	1	T3N1 R0	1	-
Patient _26	Male	cardias	N	N	-	N	N	Y	G3	1	T2N3b R0	1	-

Supplementary Table S2. Differentially expressed genes specific in tumor tissue among the 14 clusters identified by scRNA-seq. Adjusted P value (p_val_adj) < 0.05, avg log2 fold change > 0.3, MAST test.

B CELLS	CD4 Naive	CD4 Tcm	CD8 Exhausted	CD8 Tcm	CD8 Tem	CD8 Temra	MAIT	MAST	MYELOID	NK	PLASMA	PROLIFERATING T	Treg
CD79A	CCR7	NMB	CXCL13	RNF213	SRSF7	EEF1G	LTB	AL157895.1	CYBB	KLRC2	JSRP1	HMGB2	IL2RA
HLA-DRB1	EEF1A1	CHN1	CD8A	HNRNPH1	CXCR4	PLAAT4	CCL20	BTK	SOD2	TXK	SELENOM	RRM2	LTB
HLA-DPB1	EEF1B2	CXCL13	CTSW	FUS	JUND	CD52	TNFRSF25	ALDH1A1	RAB31	KIR2DL4	TP53INP1	SMC2	ARID5B
HLA-DPA1	RACK1	TOX2	GZMB	MT-ND4L	SRSF2	B2M	MYBL1	ARHGEF6	KYNU	LAT2	ST6GAL1	TUBA1B	LINC01943
HLA-DRB5	LTB	SPOCK2	NKG7	ISG15	CD81	YPEL5	TPT1	GCSAML	TIMP1	MIF	TPD52	DUT	LAIR2
IGHM	TPT1	MFHAS1	LAG3	INTS6	DNAJA1	PPDPF	EEF1B2	SLC45A3	MARCKS	HCST	MEF2C	PKMYT1	RTKN2
CD74	EEF1D	RBPJ	CCL5	CCNL1	CCL4L2	GZMK	GPR183	BACE2	ALDH2	MPG	COBLL1	MCM7	HTATIP2
EEF1B2	FAU	ARID4B	CD8B	SMCHD1	NAA50	HLA-C	FURIN	IL18	SLC43A2	PCID2	CRELD2	RAD51AP1	ZBTB38
EZR	UBA52	JUNB	ENTPD1	USP15	DNAJB1	NDUFS5	FAM107B	AKAP12	RNASE6	ARPC2	IGHG3	CENPM	CACYBP
HLA-DMA	KLF2	CCR7	AHI1	ARGLU1	RALGAPA1	H3F3B	CD69	LEO1	IFI30	GAS5	CPNE5	DHFR	UBE2B
CD79B	GIMAP7	GSPT1	PDCD1	DDX3X	TGFB1	TUBA4A	HSPA8	FCER1A	ACTN1	EEF1G	TOR3A	CENPU	SOD1
HLA-DMB	NACA	KIAA0319L	KLRD1	TAPBP	CLDND1	CALM2	PIM1	RGS13	CD86	SNHG5	CLPTM1L	PCNA	CYTOR
LAPTM5	NOP53	CCR6	LINC01871	PPP1R10	BRD2	ITM2C	PDCD4	SWAP70	ANPEP	GSTP1	ARSA	KNL1	BIRC3
PHACTR1	TOMM7	FAM107B	RBPJ	SMG1	FABP5	MT1E	TANK	RAB32	MNDA	SNHG29	IGHG4	BRCA1	JAK1
ZFAS1	EIF3E	PLIN2	AKAP5	DYNC1H1	NASP	SNRPD2	G3BP2	RHOBTB3	FNIP2	CDC42SE1	AC012236.1	ORC6	GBP5
CTSH	EEF2	LIMA1	FKBP5	MACF1	BCLAF1	LYAR	KLF6	PBX1	HMOX1	EIF4A1	GNG7	MCM3	SMAP2
DDX21	PFDN5	NAP1L4	ETS1	NKTR	FAM133B	KRTCAP2	TTC39C	HES1	SLC15A3	CD99	SDC1	FANCI	ZNF292
REL	BTF3	MYCBP2	METRNL	SP100	MIDN	CREM	RAP1B	ABCC4	RBM47	FOS	CHPF	GINS2	RAB11FIP1
AL121944.1	HINT1	TNFRSF25	ALOX5AP	SYNE2	MYADM	ZFP36	AC058791.1	CATSPER1	S100A12	AC020916.1	SRM	HELLS	LAYN
RNASET2	SNHG8	TNFAIP8	SYTL3	N4BP2L2	ADGRE5	MIF	ANKRD28	AC106739.1	KCTD12	SERTAD1	RALGPS2	CENPH	UCP2
UPF2	COMMD6	TCF7	NR3C1	LY6E	JPT1	GZMM	ZNF267	ARMH1	SMIM25	CCND3	CPEB4	NUDT1	TRAF3
EIF1AY	EEF1G	STK17A	HAVCR2	SLC38A2	ITM2C	MT2A	ITM2B	NTRK1	ASPH	BIN1	IGHG1	NDC80	PMAIP1
SNX3	GPR171	TMEM173	VCAM1	YME1L1	IFRD1	DDIT4	ANKRD12	NT5DC2	DENND5A	LSP1	DNAAF1	ATAD2	PAK2
MTRNR2L8	UXT	ITGA4	NAP1L4	TAF1D	PIP4K2A	RNASEK	TNFAIP8	FER	LILRB4	TGFB1	AC007952.4	SGO1	PHTF2
NR4A1	ARID5A	PHACTR2	CEBPB	NSD3	NEU1	APOBEC3G	MYADM	P2RX1	BCAT1	ECH1	BHLHE41	KIF11	ZC3H7A
ALG13	TMEM123	NCOA7	CEMIP2	PARP14	KLF2	PLP2	STK17A	ST8SIA6	FGD4	ADGRE5	MIR29B2CHG	FEN1	NDUFV2
CD48	ZFAS1	TLK1	IFI6	DHX36	SMCHD1	BLOC1S1	GNG2	GMPR	MAP3K20	IER2	IGHG2	MYBL2	CDKN1B
MTRNR2L12	OCIAD2	FAM118A	JAML	SPTAN1	POLR2A	PRR13	MZT2A	CADPS	IL13RA1	NDUFS8	GAS6	WDR34	ZNRF1
SELL	CRIP1	ZNRF1	LAYN	STAT4	SMARCA5	GLIPR1	PBXIP1	ST3GAL4	PLA2G7	ABHD17A	ST6GALNAC4	MCM5	CAST
PLCG2	MBP	CCDC6	ITGA4	TLE4	H2AFX	PDCL3	LPIN2	CD33	SLC7A7	SELENOH	ITGA6	RRM1	SELL
CTSZ	TXNIP	AGFG1	GABARAPL1	ACTN4	STK17A	PSMB9	PMEPA1	TMEM255B	MCEMP1	EFHD2	CLIC4	MCM4	RAP1A
TMEM243	EIF3L	STAT1	CHN1	IRF1	AC245297.3	DHRS7	EML4	CD22	SIRPA	SLC16A3	TXNDC11	PHF19	FOXP1
ATP2B1	SMDT1	FOXO1	HLA-DQA2	EML4	KIAA1551	SS18L2	NDUFV2	TMEM233	LILRA5	TLE4	PDK1	LIG1	GADD45A
TRIM38	RNASET2	EPSTI1	CLEC2D	FLNA	MXD4	TENT5C	GPR65	CDK15	ADM	NR4A2	HSH2D	PRC1	GLRX
SP110	FAM107B	CDK6	ZBTB1	C6orf62	AKIRIN1	OCIAD2	NOP53	ELSPBP1	CD36	CD96	CADM1	CHEK1	CREBRF
NR4A2	PLP2	MSI2	FAM118A	PRPF4B	CNOT2	GABARAP	MT-ND6	ITGA2B	SEMA6B	NEU1	CHST2	RFC4	PRDM2
BOD1L1	PRDX2	TMEM123	KRT86	KANSL1	HCG18	TBC1D10C	STK24	HS3ST1	CDC42EP2	IFRD1	CFL2	KIF23	MALT1
RILPL2	LMNA	GRAMD1A	CBLB	PTPN7	TUBA1C	CD69	AES	SPECC1	SLC16A10	PIP4K2A	PGM3	HIRIP3	RNF145
GRB2	S1PR4	POR	MX1	POLR2A	DEDD2	VPS37B	CLEC2B	RAB34	IL3RA	CNOT2	PECAM1	SKA2	PRDX2
BACH1	MZT2A	SFXN1	SLC7A5	JMJD6	MARCKSL1	FABP5	SATB1	SDSL	RAB20	NSMCE1	EIF2AK3	SNRNP25	TFRC
PDE7A	ARL4A	CUL3	ZFP36L2	NCOR1	AC016831.7	FKBP11	AC016831.7	RENBP	TNFAIP6	OSTF1	OTUD1	GMNN	BCL2
TTC3		ZEB1	LYST	PSMA3-AS1	TAGAP		CDC42EP3	VAT1		DHRS7	HSPA6	BRCA2	TLK1
PHF20		MINOS1	SRGAP3	WAC	ORAI1		AHNAK	RAB37		CCND2	PLCG2	WDR76	TTC39C
ANKRD37		PPP1CC	RDH10	RUNX3	CRYBG1		ORAI1	LINC00672		POLR2K		ACYP1	PIM2
		TIFA	BPTF	CXCR4	ODC1		ARL4A	CSF2RB		PSMD13		AL441992.1	RYBP
		PSIP1	NFIL3	AC016831.7	INTS6		EMB			DGUOK		NCAPH2	SERINC3
		ITGAL	KIR2DL4	AKNA	NR4A2		PNP			RNASEK		NCAPD3	TMEM173
		STAT5B	EIF2AK2	CSNK1D	RBM38		SEPT6			AC245297.3		HADH	BACH1
		SLFN5	IFI44L	AAK1	LBH		GPR171					IFI27L1	TNFRSF25
		ZNF281	KLRC2	RBBP6	PDE3B		UBE2S						SDF4

Supplementary Table S3. List of all antibodies for flow cytometry used in this study. Information about the staining protocol is also included. Table legend: brilliant blue (BB), brilliant ultraviolet (BUV), brilliant violet (BV), room temperature (RT).

Specificity	Fluorochrome	Clone	Manufacturer	Cat. #	Included in mix	Incubation time
Zombie	BV510	-	Biolegend	423102	viability staining mix (RT)	20 min, RT
CCR7	BB660	150503	BD	625454	chemokine receptor staining mix (37°)	20 min, 37°
CXCR6	PE-Cy7	K041E5	Biolegend	356012	chemokine receptor staining mix (37°)	20 min, 37°
CD4	BUV615	SK3		624297	surface staining mix (RT)	20 min, RT
CD39	APC-H7	EBioA1	eBioscience	47-0399-41	surface staining mix (RT)	20 min, RT
CD8	BUV805	SK1	BD	564912	surface staining mix (RT)	20 min, RT
CD28	BV786	CD28.2	BioLegend	302950	surface staining mix (RT)	20 min, RT
CD45RO	BV570	UCHL1	BioLegend	304226	surface staining mix (RT)	20 min, RT
CD161	BV605	HP-3G10	Biolegend	339915	surface staining mix (RT)	20 min, RT
PD1	BV480	EH12.1	BD	566112	surface staining mix (RT)	20 min, RT
CD25	BUV563	2A3	BD	612918	surface staining mix (RT)	20 min, RT
CD69	BUV737	FN50	BD	564439	surface staining mix (RT)	20 min, RT
CD103	BV421	Ber-Act8	Biolegend	350214	surface staining mix (RT)	20 min, RT
CD127	PE-Cy5	eBioRDR5	eBioscience	15-1278-42	surface staining mix (RT)	20 min, RT
CD45	BUV395	HI30	BD	563792	surface staining mix (RT)	20 min, RT
CD38	BV711	HIT2	BioLegend	303528	surface staining mix (RT)	20 min, RT
HLADR	BUV661	G46-6	BD	565073,0	surface staining mix (RT)	20 min, RT
GZMK	PE	GM6C3	SantaCruz	sc-56125	Intracellular staining mix (4°)	30 min, RT
KI67	FITC	B56	BD	556026	Intracellular staining mix (4°)	30 min, 4°
CD3	BUV496	UCHT1	BD	612940	Intracellular staining mix (4°)	30 min, 4°
GZMB	APCR700	GB11	BD	560213	Intracellular staining mix (4°)	30 min, 4°
BATF	APC	MBM7C7	eBioscience	BMS50-9860-42	Intracellular staining mix (4°)	30 min, 4°

Table legend
BB: brilliant blue
BUV: brilliant ultraviolet
BV: brilliant violet
RT: room temperature

Supplementary Table S4. List of all the genes found to have gain or loss in CNV with SODEGIR analysis. Information about the genes that reported to have gain or loss in CNV after SODEGIR analysis. In yellow are highlighted the genes that were found to be also IPA biomarkers.

GAIN			
chr	start	end	symbol
chr1	2025043	2030758	GABRD
chr3	172039578	172401669	FNDC3B
chr5	10275875	10307902	CMBL
chr5	13690331	13944543	DNAH5
chr5	16473038	16545048	RETREG1
chr6	41033058	41039221	UNC5CL
chr6	41158506	41163186	TREM2
chr6	41267926	41286682	TREM1
chr6	41736711	41754109	PGC
chr6	44000580	44007612	C6orf223
chr7	897900	955407	ADAP1
chr7	2631986	2664802	TTYH3
chr7	16791811	16833433	AGR2
chr7	16859412	16881987	AGR3
chr7	26291895	26374329	SNX10
chr7	27170592	27180261	HOXA10
chr7	29563835	29567293	PRR15
chr7	37905932	38025695	SFRP4
chr7	41667168	41705834	INHBA
chr7	93188534	93226469	HEPACAM2
chr7	94394895	94431227	COL1A2
chr8	69563769	69660915	SULF1
chr8	70669339	70790371	XKR9
chr8	71841549	71844468	MSC
chr8	75407914	75566834	HNF4G
chr8	94127162	94217303	CDH17
chr12	24810024	24949101	BCAT1
chr12	26120030	26125037	BHLHE41
chr12	31382226	31591136	DENND5B
chr12	69348381	69354234	LYZ
chr12	69825227	69959097	MYRFL
chr12	70638073	70779405	PTPRR
chr13	37562583	37598844	POSTN
chr13	37636636	37870425	TRPC4
chr13	38687077	38887131	FREM2
chr13	110148963	110307157	COL4A1

LOSS			
chr	start	end	symbol
chr1	22117313	22130641	WNT4
chr17	18377778	18389647	EVPLL
chr17	18968789	19004764	FAM83G
chr17	19648136	19685760	ALDH3A2
chr17	19737984	19748943	ALDH3A1
chr18	63897174	63936111	SERPINB10
